# Pushing the needle beyond preload: toward dynamic evaluation of venous congestion

**DOI:** 10.62675/2965-2774.20260411

**Published:** 2026-06-02

**Authors:** Rafael Hortêncio Melo, Eduardo Kattan, Luciano César Pontes de Azevedo

**Affiliations:** 1 Hospital Israelita Albert Einstein São Paulo SP Brazil Hospital Israelita Albert Einstein - São Paulo (SP), Brazil.; 2 Pontificia Universidad Católica de Chile Facultad de Medicina Departamento de Medicina Intensiva Santiago Chile Departamento de Medicina Intensiva, Facultad de Medicina, Pontificia Universidad Católica de Chile - Santiago, Chile.

## INTRODUCTION

Hemodynamic optimization in critical care has long relied on the concept of fluid responsiveness – the ability of the heart to increase stroke volume in response to a volume challenge. Dynamic indices, such as pulse pressure variation and passive leg raising, predict this response.^(1)^ However, only about half of critically ill patients are fluid responsive,^(2)^ and venous congestion may coexist even in responders,^(3)^ reflecting limited right-heart or venous compliance. In such cases, further volume expansion may raise venous pressures, promote interstitial fluid accumulation, and precipitate organ dysfunction.^(4)^ This highlights the importance of incorporating venous and interstitial physiology into hemodynamic evaluation to assess the capacity of the circulation to accommodate fluid without generating excessive venous pressure.

## INSIGHTS FROM VOLUME KINETICS

**Figure 1:**
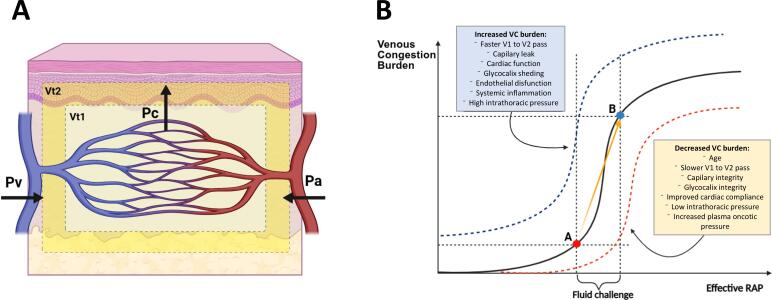
Mechanisms and conceptual framework of venous congestion. (A) Microvascular forces and interstitial compartments driving venous congestion. Arteriolar tone and arterial pressure determine capillary hydrostatic pressure, while venous pressure raises downstream resistance and limits drainage. Infused fluid first distributes between plasma and the fast-exchange interstitium; once saturated, excess volume shifts to the slow-exchange interstitium, promoting edema. (B) Conceptual curve illustrating venous congestion burden versus effective right atrial pressure. Congestion rises sharply once the lymphatic reserve is exhausted and interstitial compliance increases (near 0mmHg). Patients may remain fluid responsive yet not fluid tolerant if post-bolus pressures exceed this inflection, reflected by worsening venous Doppler patterns (e.g., increased portal pulsatility or discontinuous intrarenal flow). Created with Scientific Image and Illustration Software (BioRender, https://biorender.com).

Volume-kinetic studies have elucidated the fate of infused crystalloids. Following administration, fluid equilibrates between the plasma and a fast-exchange interstitial compartment (Vt1). Since this compartment expands by approximately 600 - 800mL - corresponding to an infusion of roughly 1.3 - 1.5L of crystalloid over 30 minutes - subsequent fluid distributes to a slow-exchange interstitial compartment (Vt2), which serves as a reservoir for sustained interstitial fluid retention and edema.^(5)^ The threshold for this transition is not constant: it increases under general anesthesia or hypotension, when interstitial fluid redistributes toward the plasma, and decreases during inflammatory states in which cytokine-mediated reductions in interstitial pressure accelerate tissue sequestration. Crystalloid solutions promote this transition earlier, whereas hyperoncotic colloids such as 20% albumin have traditionally been assumed to counterbalance interstitial accumulation by recruiting fluid back into the vascular space.^(6)^ However, alternative models of microvascular fluid exchange based on the revised Starling equation and the endothelial glycocalyx suggest that raising plasma oncotic pressure mainly reduces further filtration rather than producing clinically meaningful reabsorption of interstitial fluid, which may explain why albumin therapy has not consistently prevented or reversed tissue edema in clinical studies.^(7)^ Beyond this transition, as cumulative infusion volumes increase, a progressively smaller fraction of the administered fluid remains intravascular, and an increasing proportion becomes sequestered in the interstitium. In critical illness, endothelial barrier dysfunction and capillary leak - frequent in sepsis and systemic inflammation - further reduce interstitial pressure, increase permeability, and impair lymphatic drainage.^(8,9)^ These combined mechanisms may partially explain the association between positive fluid balance and adverse outcomes, including increased mortality, prolonged mechanical ventilation, and higher risk of acute kidney injury.^(10,11)^ The dynamic interaction between distributional physiology (Vt1 → Vt2) and endothelial integrity ultimately delineates how apparently therapeutic plasma expansion can evolve into pathologic venous congestion and tissue edema (Figure 1A). Understanding this redistribution provides a physiologic substrate for interpreting venous congestion not merely as fluid excess, but as the hemodynamic expression of interstitial-venous coupling. This pathophysiologic continuum mirrors current perspectives on fluid stewardship in surgical critical care, which emphasize the need to balance resuscitation against the risk of fluid accumulation syndrome and glycocalyx disruption.^(12)^

Vt2 - slow-exchange interstitial compartment; Vt1 - fast-exchange interstitial compartment; Pc - capillary hydrostatic pressure; Pa - arterial pressure; Pv - venous pressure; VC - venous congestion; RAP - right atrial pressure.

## FROM VOLUME KINETICS TO VENOUS CONGESTION PHENOTYPES

Congestion is not a uniform process but a spectrum of pressure-volume-function interactions. Guinot et al.^(13)^ described three overlapping endotypes:

– Hemodynamic congestion: elevated filling pressures without major interstitial accumulation.
– Volemic congestion: positive fluid balance and tissue edema despite normal pressures.
– Systemic congestion: biventricular dysfunction with high venous pressures and Doppler abnormalities.

Beyond taxonomy, these endotypes suggest different therapeutic priorities at the bedside. In hemodynamic congestion, the main goal is to lower venous pressures by modulating right ventricular afterload and heart-lung interactions - for example, adjusting pulmonary vascular tone and inotropy - rather than simply removing fluid. Volemic congestion, in contrast, calls for net negative fluid balance and avoidance of further fluid loading in patients who are already volume replete. In systemic congestion, where biventricular dysfunction and severely abnormal venous Doppler patterns coexist, management often requires a combined strategy of decongestion, cardiac function optimization, and close monitoring of venous congestion indices to prevent further organ injury.

These clinical profiles are also consistent with what volume kinetics predicts about the relationship between venous pressure and interstitial fluid. Venous hypertension and interstitial fluid accumulation may evolve independently, depending on the compliance of the venous and interstitial compartments. As interstitial (Vt2) volume expands, its hydraulic coupling with the venous system intensifies: rising venous pressure increases capillary hydrostatic pressure and promotes further filtration into the interstitium, while elevated interstitial pressure impairs lymphatic drainage. This creates a self-perpetuating cycle of capillary leak and congestion. In this context, fluid overload reflects not merely excess water, but the inability of venous and interstitial compartments to accommodate volume without converting it into pressure - and, ultimately, into organ dysfunction.

## INCORPORATION OF DYNAMIC ASSESSMENT OF VENOUS CONGESTION

Long before the modern concepts of fluid tolerance and venous congestion were formulated, clinicians already recognized the importance of limiting the hemodynamic cost of fluid loading. Central venous pressure (CVP) was among the first variables used to gauge this balance. Early protocols, such as the "5-2 rule" described by Weil et al.,^(14)^ advised interrupting fluid infusion when CVP increased by more than 2cmH_2_O after a small bolus - an intuitive attempt to identify a dynamic safety threshold beyond which further volume would be deleterious. Recent studies demonstrate that even modest fluid loading can provoke measurable changes in venous flow patterns before overt clinical signs of overload. Ruste et al. showed that in ICU patients with baseline systemic congestion (inferior vena cava ≥ 20mm), a 4mL/kg crystalloid bolus significantly increased both CVP and VExUS congestion scores, without improving perfusion indices such as lactate or capillary refill.^(15)^ Similarly, Morosanu et al. observed that small volume infusions after cardiac surgery induced transient portal and intrarenal venous congestion, evidenced by a rise in portal vein pulsatility above 50% and the appearance of discontinuous intrarenal venous flow.^(16)^ Notably, these authors went one step further. They were able to identify patients at risk of venous congestion through a passive leg raising maneuver, providing a highly sensitive and specific tool to identify patients at risk of venous congestion, without actually exposing them to potentially deleterious fluid administration.

Together, these findings confirm that venous congestion is a dynamic, continuous, and measurable process. Doppler interrogation of venous system provides real-time insight into how venous pressure propagates through the circulation, integrating the effects of right-heart function, mean systemic filling pressure, and organ compliance (Figure 1B) These findings might partly explain why tissue perfusion sometimes fails to improve or even worsens despite increases in cardiac output following fluid administration, a phenomenon known as hemodynamic incoherence or decoupling.^(16,17)^

## CONCLUSION

The transition from assessing preload responsiveness to evaluating venous tolerance represents a logical progression in fluid therapy. Integrating fluid kinetics, congestion phenotyping, tissue perfusion, and real-time venous monitoring could establish a more complete framework of fluid tolerance, identifying not only who benefits from fluid administration but also who is at risk of adverse effects.

## Data Availability

Not applicable. This manuscript is a narrative viewpoint article and does not report original datasets, software code, or other research materials requiring public availability.
